# Relating Structure and Function in the Human Brain: Relative Contributions of Anatomy, Stationary Dynamics, and Non-stationarities

**DOI:** 10.1371/journal.pcbi.1003530

**Published:** 2014-03-20

**Authors:** Arnaud Messé, David Rudrauf, Habib Benali, Guillaume Marrelec

**Affiliations:** Laboratoire d'Imagerie Fonctionnelle, UMR678, Inserm/UPMC Univ Paris 06, Paris, France; Hamburg University, Germany

## Abstract

Investigating the relationship between brain structure and function is a central endeavor for neuroscience research. Yet, the mechanisms shaping this relationship largely remain to be elucidated and are highly debated. In particular, the existence and relative contributions of anatomical constraints and dynamical physiological mechanisms of different types remain to be established. We addressed this issue by systematically comparing functional connectivity (FC) from resting-state functional magnetic resonance imaging data with simulations from increasingly complex computational models, and by manipulating anatomical connectivity obtained from fiber tractography based on diffusion-weighted imaging. We hypothesized that FC reflects the interplay of at least three types of components: (i) a backbone of anatomical connectivity, (ii) a stationary dynamical regime directly driven by the underlying anatomy, and (iii) other stationary and non-stationary dynamics not directly related to the anatomy. We showed that anatomical connectivity alone accounts for up to 15% of FC variance; that there is a stationary regime accounting for up to an additional 20% of variance and that this regime can be associated to a stationary FC; that a simple stationary model of FC better explains FC than more complex models; and that there is a large remaining variance (around 65%), which must contain the non-stationarities of FC evidenced in the literature. We also show that homotopic connections across cerebral hemispheres, which are typically improperly estimated, play a strong role in shaping all aspects of FC, notably indirect connections and the topographic organization of brain networks.

## Author Summary

By analogy with the road network, the human brain is defined both by its anatomy (the ‘roads’), that is, the way neurons are shaped, clustered together and connected to each others and its dynamics (the ‘traffic’): electrical and chemical signals of various types, shapes and strength constantly propagate through the brain to support its sensorimotor and cognitive functions, its capacity to learn and adapt to disease, and to create consciousness. While anatomy and dynamics are organically intertwined (anatomy contributes to shape dynamics), the nature and strength of this relation remain largely mysterious. Various hypotheses have been proposed and tested using modern neuroimaging techniques combined with mathematical models of brain activity. In this study, we demonstrate the existence (and quantify the contribution) of a dynamical regime in the brain, coined ‘stationary’, that appears to be largely induced and shaped by the underlying anatomy. We also reveal the critical importance of specific anatomical connections in shaping the global anatomo-functional structure of this dynamical regime, notably connections between hemispheres.

## Introduction

Coherent behavior and cognition involve synergies between neuronal populations in interaction [1]–[3]. Even at rest, in the absence of direct environmental stimulations, these interactions drive the synchronization of spontaneous activity across brain systems, shedding light on the large-scale anatomo-functional organization of the brain [4]. The study of such patterns of synchronization has known important developments due to recent methodological advances in brain imaging data acquisition and analysis. These advances have enabled investigators to estimate interactions in the brain by measuring functional connectivity (FC) from resting-state functional MRI (rs-fMRI). Analyses of FC at rest have supported the hypothesis that the brain is spatially organized into large-scale intrinsic networks [5]–[7], e.g. the so-called resting-state networks [8], [9], such as the default mode network, which have been linked to central integrative cognitive functions [10]–[13]. The study of large-scale intrinsic networks from rs-fMRI has become a central and active area for neuroscience research. However, the mechanisms and factors driving FC, as well as their relative contribution to empirical data, are still highly debated [14] and remain to be elucidated.

Theoretical rationale and empirical findings support the hypothesis that FC is driven and shaped by structural connectivity (SC) between brain systems, i.e., by the actual bundles of white matter fiber connecting neurons [15]. As a first approximation, SC can be inferred from fiber tractography based on diffusion-weighted imaging (DWI) [16]–[19]. A recent study [20], which focused on a small subset of robustly estimated structural connections, demonstrated the existence of a statistical, yet complex, correspondence between FC and specific features of SC (e.g., low vs. high fiber density, short vs. long fibers, intra vs. interhemispheric connections). However, a large part of FC cannot be explained by SC alone [21]. There appears that FC is the result of at least two main contributing factors: (i) the underlying anatomical structure of connectivity, and (ii) the dynamics of neuronal populations emerging from their physiology [3]. A key issue is to better understand the relative contributions of these two components to FC. Besides, recent studies using windowed analyses have suggested that FC estimated over an entire acquisition session (referred to as ‘stationary FC’ in the literature) breaks down into a variety of reliable correlation patterns (also referred to as ‘dynamic FC’ or ‘non-stationarities’) when estimated over short time windows (30 s) [14], [22]. The authors advocated that FC estimated over short time windows (or windowed FC, for short) mostly reflects recurrent transitory patterns that are aggregated when estimating FC over a whole session. They further suggested that whole-session FC may only be an epiphenomenon without clear physiological underpinning, and not the reflection of an actual process with stationary FC [14]. This perspective remains to be reconciled with the fact that whole-session FC has been found to be highly reproducible, functionally meaningful and a useful biomarker in many pathological contexts [23], [24]. Note that, in the recent literature of fMRI data analysis, stationarity implicitely refers to a stationary FC (i.e., the invariance of FC over time), to be contrasted with the more general notion of (strong) stationarity, where a model or process is stationary if its parameters remain constant over time [25], [26]. SC being temporally stable at the scale of a whole resting state fMRI session (typically 10 min), we could expect SC to drive a stationary process (in the strong sense). Since SC is furthermore expected to drive FC, we can hypothesize that this stationary process contributes to generate a stationary FC.

In order to bring together the structural and dynamical components underlying FC, some studies have used computational models that incorporate SC together with biophysical models of neuronal activity to generate coherent brain dynamics [27]–[32]. This approach has yielded promising results for the understanding of the relationship between structure and function [17], [33], [34]. Here, we used a testbed of well-established generative models simulating neuronal dynamics combined with empirical measures, to investigate the relative contributions of anatomical connections, stationary dynamics, and non-stationarities to the emergence of empirical functional connectivity. In particular, we considered the following hypotheses: (H1) part of FC directly reflects SC; (H2) models of physiological mechanisms added to SC increase predictive power all the more as they are complex; (H3) part of the variance of FC that is unexplained by models is due to issues in the estimation of SC, e.g., problems with measuring homotopic connections; (H4) there is an actual stationary process reflected in whole-session FC that is not merely an artifact but substantially reflects the driving of the dynamics by SC.

In order to test these hypotheses and estimate the relative contribution of anatomy, stationary dynamics and non-stationarities to FC, we relied on the following approach. After *T*_1_-weighted MRI based parcellation of the brain (*N* = 160 regions), SC was estimated using the proportion of white matter fibers connecting pairs of regions, based on probabilistic tractography of DWI data [35]. FC was measured on rs-fMRI data using Pearson correlation between the time courses of brain regions. We quantified the correlation between SC alone and FC as a reference, and also fed SC to generative neurodynamical models of increasing complexity: a spatial autoregressive (SAR) model [36], analytic models with or without conduction delays [28]–[31], [37], and biologically constrained models [29], [32]. Importantly, all these models were used in their stationary regime in the strong sense, since their parameters were not changed during the simulations. Of these models, only the SAR is explicitely associated with a stationary FC; other, more complex models, generate dynamics that are compatible with a non-stationary FC. We computed FC from data simulated by these models and compared the results to empirical FC. For each model, performance was quantified using predictive power [29], for each subject as well as on the ‘average subject’ (obtained by averaging SC and empirical FC across subjects). Values for the model parameters were based on the literature, except for the structural coupling strength that was optimized in order to maximize each model's performance.

## Results

### Predictive power of models

**Figure 1:**
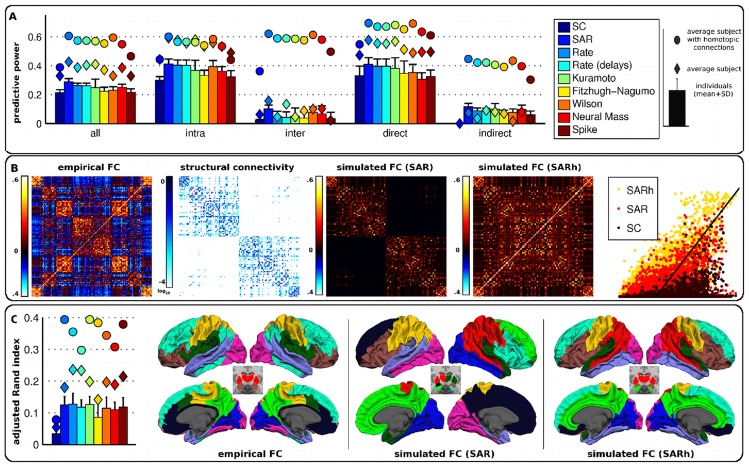
Performance of computational models. (A) Predictive power for all connections and when restricted to intra/interhemispheric, direct/indirect connections. For each type of connections and each model, we represented the individual predictive powers (bar chart representing means and associated standard deviations), as well as the predictive power for the average subject computed using the original SC (diamonds), or after adding homotopic connections (circles). Of note, SC alone has no predictive power (zero) for the subset of indirect connections, by definition. (B) Patterns of SC, empirical FC and FC simulated from the SAR model for the average subject and associated scatter plot of simulated versus empirical FC (solid line represents perfect match). SARh stands for the SAR model with added homotopic connections. Matrices were rearranged such that network structure is highlighted. Homologous regions were arranged symmetrically with respect to the center of the matrix; for instance, the first and last regions are homologous. (C) Similarity of functional brain networks across subjects (bar chart with means and associated standard deviations), for the average subject (diamonds), and when adding homotopic connections (circles) (left). Network maps for the average subject and empirical FC, as well as for FC simulated using either the SAR model with original SC or the SARh.

In agreement with H1, SC explained a significant amount of the variance of whole-session FC for all subjects, as did all generative models (permutation test, *p*<0.05 corrected) (Figure 1, panel A). Generative models predicted FC better than SC alone (paired permutation test, *p*<0.05 corrected). Predictive power obtained with the average subject ranged from 0.32 for SC alone to 0.43 for the SAR model (Table 1). For a given model, predictive power was reproducible across subjects. Contrary to our hypothesis H2, generative models had similar performance, and complexity was not predictive of performance. The results remained unchanged when no global signal regression was applied (Figure S1). Also, findings were found to be similar for SC alone and the SAR model at finer spatial scales (*N* = 461 and *N* = 825 regions, Figure S2) and consistent with a replication dataset (Figure S3). Most importantly, a large part of the variance (*R* ^2^) in the empirical data (at least 82%) remained unexplained by this first round of simulations.

**Table 1:** Predictive power for SC and the SAR model.

	all	direct	indirect	intra	inter
**SC**					
individuals, mean (SD)	0.21 (0.02)	0.33 (0.06)	-	0.30 (0.02)	0.02 (0.01)
average subject	0.32	0.47	-	0.44	0.06
average subject with homotopic connections	0.39	0.55	-	0.44	0.36
**SAR**					
individuals, mean (SD)	0.29 (0.02)	0.41 (0.05)	0.12 (0.02)	0.41 (0.03)	0.10 (0.03)
average subject	0.43	0.58	0.07	0.59	0.16
average subject with homotopic connections	0.61	0.69	0.45	0.60	0.62

### Role of homotopic connections

We reasoned (see hypothesis H3) that part of the unexplained variance could reflect issues with the estimation of SC from DWI, which can be expected because of limitations in current fiber tracking algorithms and the problem of crossing fibers [38]. We know for instance that many fibers passing through the corpus callosum are poorly estimated in diffusion imaging, in particular those connecting more lateral parts of the cerebral cortex [39]. Yet, the corpus callosum is the main interhemispheric commissure of the mammal brain, see [40]. It systematically connects homologous sectors of the cerebral cortex across the two hemispheres in a topographically organized manner, with an antero-posterior gradient, through a system of myelinated homotopic fibers or ‘homotopic connections’. The hypothesis of an impact of SC estimation problems on FC unexplained variance was supported by the observation that, in our results, intrahemispheric connections yielded on average a much higher predictive power (e.g., 0.59 for the SAR model) than interhemispheric connections (0.16 for the SAR model).

**Figure 2:**
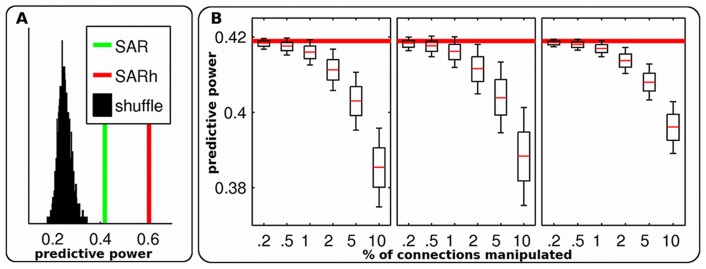
Manipulation of SC. (A) Predictive power of the SAR model with original SC (green), when adding homotopic connections (‘SARh’, red), or with shuffled homotopic connections (black). (B) Predictive power of the SAR model with original SC (red) and when SC values were randomly permuted, removed or added (from left to right). For each graph, predictive power was quantified as a function of the percentage of connections manipulated.

In order to further test the role of white matter connections in driving FC, we artificially set all homotopic connections to a constant SC value (0.5) for the average subject and reran all simulations. As a result, the predictive power strongly increased for all models (Figure 1, panels A and B), ranging from 0.39 for SC alone to 0.61 for the SAR model (Table 1). Thus the variance unexplained (1-*R* ^2^) was reduced to 63%. Moreover, predictive power for intra and interhemispheric connections became equivalent (0.60 and 0.62, respectively). Interestingly, adding homotopic connections also led to a substantial increase in predictive power for indirect connections, that is, pairs of regions for which SC is zero (increasing from 0.07 to 0.45). The effect of adding interhemispheric anatomical connections on increasing predictive power was highly specific to homotopic connections. When applying the SAR model to the SC matrix with added homotopic connections and randomly permuting (10 000 permutations) the 80 corresponding interhemispheric connections (one region in one hemisphere was connected to one and only one region in the other hemisphere), the predictive power strongly decreased, even compared to results with the original SC (Figure 2, panel A). Moreover, we further assessed the specificity of this result by systematically manipulating SC. In three different simulations, we randomly removed, added, and permuted structural connections (10 000 times). In all cases, the predictive power decreased as a function of the proportion of connections manipulated (Figure 2, panel B). Moreover, changes induced by these manipulations remained small (<0.05), far below the changes that we were able to induce by adding homotopic connections. All in all, these results suggest that homotopic connections play a key role in shaping the network dynamics, in a complex and non-trivial manner.

### Predicting functional brain networks

Beyond predicting the overall pattern of FC, we also assessed whether models could predict the empirical organization of FC into a set of intrinsic networks. Connectivity matrices were clustered into groups of non-overlapping brain regions showing high within-group correlation and low between-group correlation, and the resulting partitions into functional brain networks were compared between empirical and simulated FC using the adjusted Rand index (see Methods). Again, the SAR model tended to perform best among all computational models (Figure 1, panel C).

Without adding homotopic connections in the SC matrix, the simulated networks highly differed from the empirical networks. In particular, most networks were found to be lateralized. After adding homotopic connections, the resemblence between simulated and empirical networks greatly improved. Networks were more often bilateral and overall consistent with the topography of empirical functional networks, including somatosensory, motor, visual, and associative networks. High FC between the amygdala and ventral-lateral sectors of the prefrontal cortex was also correctly predicted by the simulations. There were nevertheless some notable differences. First, the clustering of empirical FC yielded a long-range fronto-parieto-temporal association network (Figure 1, panel C, cyan) that was not observed in simulated FC as such. Second, a parieto-temporal cluster (Figure 1, panel C, red), which was associated with thalamo-striatal networks, was predicted by simulations but was not present in the empirical data. Third, a cluster encompassing the entire cingulate gyrus and precuneus (Figure 1, panel C, green) was predicted by simulations but was broken down into more clusters in the empirical data.

### Stationary FC, non-stationary FC, and non-stationarities

The results above show that SC plays a causal role in FC, but one can still wonder what aspects of the underlying dynamics are the most directly related to this influence. A hypothesis is that SC, in combination with stable physiological processes (e.g., overall gain in synaptic transmission), drives a stationary regime of the dynamics. This hypothesis is supported by the finding that all models tested in this study, which were used in a stationary regime (in the strong sense), could explain significantly more variance than SC alone. Furthermore, the fact that the SAR could predict FC significantly better than all other models is evidence that this stationary regime is associated with stationary FC (paired permutation test, *p*<0.05 corrected).

**Figure 3:**
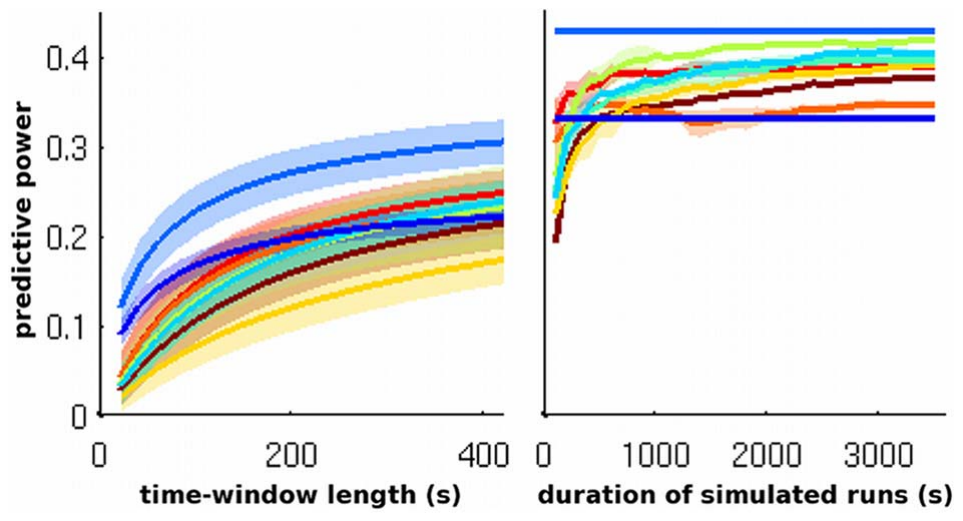
Effect of time on performance. Predictive power as a function of time-window length across subjects (left) and of duration of simulated runs on the average subject (right). For color code see Figure 1.

But, clearly, many variations in the dynamical patterns of brain activity, be it in the process of spontaneous cognition, physiological regulation, or context-dependent changes, cannot be expected to be associated with a purely stationary FC. Modeling how the brain dynamics deal with endogenous and environmental contexts should require more complex models, either stationary or non-stationary, that are able to generate non-stationary (i.e., time-varying) patterns of FC. Given that at best 37% of the variance could be explained by the model of a purely stationary FC (the SAR), we can wonder why the models of higher complexity used in our simulation testbed did not perform better in predicting FC. One possible hypothesis is that the SAR model was favored in the simulations, because we estimated FC over about 10 minutes of actual brain dynamics. In such configuration, we can imagine that the non-stationarities of FC cancel out, the estimation effectively keeping the stationary part of FC. We thus wondered whether the more complex models would better perform when non-stationary FC had the potential of being more strongly reflected in the data. We approached this question by computing predictive power on windowed FC as a function of the length of the time-window used [22], for all possible time-windows over which FC could be estimated and for all models. We also investigated the effect of simulation duration (see Methods). We found that the relative performance of more complex models was still lower than that of the SAR model (Figures 3 and S4). The average predictive power was lower for shorter time-windows and increased towards a limit for longer time-windows. The SAR model behaved like an ‘upper-bound’ for predictive power. The performance of all other models, irrespective of the size of the time-window, was between that of SC alone and that of the SAR model.

A straightforward explanation is that the non-stationary patterns of FC, as generated by the simulation models, did not match the non-stationary patterns of the empirical FC as they unfolded during the acquisition in the brain of the participants. Context-dependent and transient dynamics are likely to be missed by models of the dynamics that cannot be contextually constrained in the absence of further information. It is thus difficult to infer how much of the 63% of unexplained variance remaining in whole-session FC actually reflect physiologically meaningful non-stationary FC, and more broadly, non-stationary dynamics.

## Discussion

In the present study, we investigated the respective contributions of anatomical connections, stationary dynamics, and non-stationarities to the emergence of empirical functional connectivity. We compared the performance of computational models in modeling FC and manipulated SC in order to analyze the impact of SC on FC, with and without the filter of combined physiological models of the dynamics.

The importance of white matter fiber pathways in shaping functional brain networks is a known fact, for a review, see [15], [17], [21], [23]. Previous modeling studies have supported the importance of the underlying anatomical connections, i.e., SC, in shaping functional relationships among brain systems [16], [41], [42]. In agreement with our hypothesis H1, we showed that functional connectivity could at least in part be explained by structural connectivity alone. Adding homotopic connections in the matrix of SC, we found a slight increase in explained variance when considering the prediction of whole-session FC from SC alone (+4% of explained variance). In agreement with H2, adding models of physiological interactions above and beyond SC alone increased the explained variance in whole-session FC, by 8% for the best performing model, the SAR model, when no homotopic connections were added, and by 22% when homotopic connections were added. This latter fact, which strongly supports H3, suggests a complex interplay between anatomy as reflected by SC and physiological mechanisms in generating FC. This impact of SC manipulations on predicted FC pertained not only to direct but also to indirect connections. For indirect connections, whole-session FC was much better predicted after adding homotopic connections to SC than before adding them (0.45 versus 0.07 in predictive power). The problem of limited predictive power for FC based on SC when considering indirect connections has puzzled the field [43]. For this reasons many studies only assess the performance of models on direct connections. Here, we showed that a major factor in driving FC for indirect anatomical connections (+20% in explained variance) is the interplay between a subset of anatomical connections, i.e., homotopic connections (which are typically underestimated by DWI), and physiological parameters that generate the dynamics underlying FC, themselves conditioned by the possible interactions defined by SC.

Contrary to our expectation (see hypothesis H2), all models tended to perform similarly, irrespective of model complexity. The best performing model in most cases was the SAR model, a model of stationary FC driven by SC, with 63% of the variance remaining unexplained. It is likely that, above and beyond problems with the estimation of SC from DWI, and other incompressible sources of irrelevant noise, much of the unexplained variance in FC relates to non-stationary patterns in FC, and more generally to non-stationarities in the strong-sense. Such non-stationarities are difficult to model in the experimentally unconstrained resting-state and in the absence of further information regarding the specific parameters shaping FC. Irrespective of their complexity, computational models are only capable of generating prototypical brain activities, and not the subject-dependent activity that took place in the brain of the participants during scanning. The scientific necessity of modeling brain dynamics is hindered by such uncertainty and it will be a challenge to find solutions to approach this problem [26], [44]. Even though one objective for neuroscience is to propose generative models that are capable of generating detailed neuronal dynamics, generative models cannot be informed by this unknown context and, as a consequence, cannot generate context-dependent activity in a manner that would be predictive of empirical data, in the absence of additional measures and experimental controls. Nevertheless, and perhaps for that very reason, the study of non-stationarity in FC should become of central interest for the field, as such non-stationarities could explain much of FC (up to 63% according to our simulation results), and thus reflect critical mechanisms for neurocognitive processing.

In the absence of adequate modeling principles, determining the precise contribution of non-stationarities to the unexplained variance in FC is impossible, as other confounding sources of unexplained variance are expected. As we showed, even naive manipulations aimed at estimating the impact of the known errors in DWI-based reconstruction of homotopic connections showed that such errors could cause 20% of the unexplained variance in predicting empirical FC. How DWI and fiber tracking should be used for an optimal estimation of structural connectivity is still a topic of intense debates [45]–[48]. It is likely that part of the unexplained variance in predicting FC will be reduced as better estimates of SC become available.

The model showing the best results, the SAR model, explicitly modeled a stationary process with a stationary FC. In line with our hypothesis H4, empirical FC is likely to incorporate stationary components driven by SC. Further knowledge about this stationary process might be gained by analyzing FC computed over much longer periods of time than is commonly performed (e.g., hours versus minutes). This stationary process is itself likely to be only locally stationary, as it might be expected that slow physiological cycles, from nycthemeral cycles to hormonal cycles, development, learning and aging, will modify the parameters controlling it.

In the present study, we did not take into account the statistical fluctuations induced by the fact that the time series were of finite length. Such a finiteness entails that even a model that is stationary in the strong sense could generate sample moments that fluctuate over time. For instance, the sample sum of square of a multivariate normal model with covariance matrix Σ computed from time series of size *N* is not equal to *N*Σ but is Wishart distributed with *N*-1 degrees of freedom and scale matrix Σ. This phenomenon will artificially increase the part of variance that cannot be accounted for by stationary models and, hence, play against stationary models. Since it is conversely very unlikely for a non-stationary model to generate sample moments that are constant over time, statistical fluctuations cannot at the same time artificially increase the part of variance that can be accounted for by stationary models. As a consequence, not considering these statistical fluctuations made us underestimate the part of variance that can be accounted for by models that are stationary in the strong sense. In other words, our estimate of the part of variance accounted for by a stationary model is a lower bound for the true value. We can therefore be confident that taking statistical fluctuations into account will only strengthen H4.

Our goal here was to investigate how current generative models of brain acticity fare in predicting the relationship between structure and function. The complexity of some of these models was such that the simulations included here were only possible thanks to a computer cluster. The behavior of all these models depends on the values of some parameters and, in the present study, we set these parameters in agreement with the literature. In what measure this choice affects how well models predict FC is unclear. Yet a full investigation of this issue remains beyond the scope of this study, since parameter optimization through extensive exploration of the parameter space for all models is at this stage unrealistic. Nevertheless, in order to get a sense of the sensitivity of our results to parameter values in a way that is compatible with the computational power available, we explored the behavior of the Fitzhugh-Nagumo, Wilson and Kuramoto models over a subset of the parameter space (see Figures S5 and S6). We found that parameter values had little influence on predictive power, which, in all cases, remained below that of the SAR, the simplest model tested.

We formulated H2 to test for the existence of a relationship between complexity and realism in the models that we used. Indeed, there should exist a very tight connection between the two, since the more complex generative models in our study have been designed to take biophysical mechanisms into account, with parameters that are physiologically relevant and values often chosen based on prior experimental results. Now, realism usually comes at the cost of complexity. As a consequence, it is often (implicitely) assumed that, among the models we selected, the more complex a model is, the more realistic it will also be and the better it will fit the data. This is the reason why we stated H2, based on such rationale inspired from the literature, in order to put such hypothesis to the test. The results show that for the models we used, with their sets of parameters, an increase in complexity was not associated with an increase in performance. This suggests that, for these models, complexity and realism are not quite as tightly connected as expected.

Given that the SAR model is the only model that does not include a step of hemodynamic modeling (Balloon-Windkessel), it cannot be ruled out that the superiority of the SAR reflects issues with this step. In order to check that this is not the case, we computed predictive power for all models without the hemodynamic model. The predictive power was largely insensitive to the presence of the hemodynamic model (see Figure S7). In particular, the SAR model remained overall an upper bound in terms of predictive power.

Finally, we should note that we relied on a definition of SC restricted to the white matter compartment. Although this is standard in the field, in reality, local intrinsic SC exists in the gray matter. However, current models generally make prior assumptions about such SC. Moreover, intrinsic SC currently remains impossible to measure reliably for the entire brain.

In spite of the complexity of the problems and the limitations of current modeling approaches, computational modeling of large-scale brain dynamics remains an essential scientific endeavor. It is key to better understand generative mechanisms and make progress in brain physiology, physiopathology and, more generally, theoretical neuroscience. It is also central to the endeavor of searching for accurate and meaningful biomarkers in aging and disease [49]. Moreover, computational modeling of FC opens the possibility of making inference on specific biophysical parameters, including inference about the underlying anatomical connectivity itself. In spite of their limited predictive powers, simpler models can be useful in this context. The SAR model, introduced in [36], may appear well-suited to model essential stationary aspects of the generative mechanisms of FC. One interest of such a simple and analytically tractable model is that, beyond its very low computational burden, it could be the basis for straightforward estimation of the model parameters that can be used to compare clinical populations, and could constitute a potentially important biomarker of disease.

## Methods

### Ethics statement

All participants gave written informed consent and the protocol was approved by the local Ethics Committee of the Pitié-Salpêtrière Hospital (number: 44-08; Paris, France).

### Data

Twenty-one right-handed healthy volunteers were recruited within local community (11 males, mean age 22±2.4 years). Data were acquired using a 3 T Siemens Trio TIM MRI scanner (CENIR, Paris, France). For acquisition and preprocessing details, see Text S1. For each subject, the preprocessing yielded three matrices: one of SC, one with the average fiber lengths, and one of empirical FC. These matrices were also averaged across subjects (‘average subject’).

### Simulations

We used eight generative models with various levels of complexity: the SAR model, a purely spatial model with no dynamics that expresses BOLD fluctuations within one region as a linear combination of the fluctuations in other regions; the Wilson-Cowan system, a model expressing excitatory and inhibitory neuronal populations activity; the two rate models (with or without conduction delays), simplified versions of the Wilson-Cowan system obtained by considering exclusively the excitatory population; the Kuramoto model, which simulates neuronal activity using oscillators; the Fitzhugh-Nagumo model, which aims at reproducing complex behaviors such as those observed in conductance-based models; the neural-mass model, also based on conductance and with strong biophysiological constraints; and finally, the model of spiking neurons, the most constrained model in the current study which models neuron populations as attractors. For more details, see Text S2.

All models took an SC matrix as input, and all but the SAR were taken as models of neuronal (rather than BOLD) activity. Simulated fMRI BOLD signal was obtained from simulated neuronal activity by means of the Balloon-Windkessel hemodynamic model [50], [51]. Global mean signal was then regressed out from each region's time series. Finally, simulated FC was computed as Pearson correlation between simulated time series. For the SAR model, we directly computed simulated FC from the analytical expression of the covariance, see Equation (2) in Text S2. All models had a parameter that represents the coupling strength between regions. This parameter was optimized separately for each model on the average subject to limit computational burden (Text S2). After optimization, we generated three runs of 8 min BOLD activity and averaged the corresponding FCs to obtain the simulated FC for each dynamical model and each subject. For the average subject, simulated FC was obtained by feeding the average SC matrix to the different models.

### Performance

Modeling performance was assessed using predictive power and similarity of spatial patterns. Predictive power was quantified for each subject and for the average subject by means of Pearson correlation between simulated and empirical FC [29]. Regarding the similarity of functional brain networks, SC, empirical FC and simulated FC were decomposed into 10 networks using agglomerative hierarchical clustering and generalized Ward criterion [52]. The resulting networks from SC and simulated FC were compared to the ones resulting from empirical FC using the adjusted Rand index [53], [54]. The Rand index quantifies the similarity between two partitions of the brain into networks by computing the proportion of pairs of regions for which the two partitions are consistent (i.e., they are either in the same network for both partitions, or in a different network for both partitions). The adjustment accounts for the level of similarity that would be expected by chance only.

### Analysis of dynamics

Empirical and simulated windowed FC were computed on individual subjects using sliding time-windows (increment of 20 s) of varying length (from 20 to 420 s by step of 20 s). Predictive power was computed as the correlation between any pair of time-windows of equal length corresponding to simulated and empirical windowed FC, respectively. This approach was only applied to the dynamical models; for SC alone and the SAR model, simulated FC remained, by definition, constant through time and, as a consequence, windowed FC was equaled to whole-session FC. The influence of simulated run duration on predictive power was also investigated. For each model, three runs of one hour were simulated on the average subject. Predictive power was then computed as a function of simulated run duration. For the same reason as above, SC alone and the SAR model did not depend on simulation duration.

## Supporting Information

Figure S1 **Performance of computational models when no global signal regression was performed.** (A) Predictive power for all connections and when restricted to intra/interhemispheric, direct/indirect connections. For each type of connections and each model, we represented the individual predictive powers (bar chart representing means and associated standard deviations), as well as the predictive power for the average subject computed using the original SC (diamonds), or after adding homotopic connections (circles). Of note, SC alone has no predictive power (zero) for the subset of indirect connections, by definition. (B) Patterns of SC, empirical FC and FC simulated from the SAR model for the average subject and associated scatter plot of simulated versus empirical FC (solid line represents perfect match). SARh stands for the SAR model with added homotopic connections. Matrices were rearranged such that network structure is highlighted. Homologous regions were arranged symmetrically with respect to the center of the matrix; for instance, the first and last regions are homologous. (C) Similarity of functional brain networks across subjects (bar chart with means and associated standard deviations), for the average subject (diamonds), and when adding homotopic connections (circles) (left). Network maps for the average subject and empirical FC, as well as for FC simulated using either the SAR model with original SC or the SARh.
(EPS)

Figure S2 **Performance of SC alone and the SAR model at finer spatial scales.** Predictive power for all connections and when restricted to intra/interhemispheric, direct/indirect connections. For each type of connections and each model, we represented the individual predictive powers (bar chart representing mean and associated standard deviation), as well as the predictive power of the average subject computed using the original SC (diamonds), or after adding homotopic connections (circles).
(EPS)

Figure S3 **Performance of computational models on the replication dataset.** The replication dataset was from the study of Hagmann and colleagues [55]. Brain network was defined at low anatomical granularity (N = 66 regions), and connectivity measures were averaged over five healthy volunteer subjects. (A) Predictive power for all connections and when restricted to intra/interhemispheric, direct/indirect connections. For each type of connections and each model, we represented the individual predictive powers (bar chart representing means and associated standard deviations), as well as the predictive power for the average subject computed using the original SC (diamonds), or after adding homotopic connections (circles). Of note, SC alone has no predictive power (zero) for the subset of indirect connections, by definition. (B) Patterns of SC, empirical FC and FC simulated from the SAR model for the average subject and associated scatter plot of simulated versus empirical FC (solid line represents perfect match). SARh stands for the SAR model with added homotopic connections. Matrices were rearranged such that network structure is highlighted. Homologous regions were arranged symmetrically with respect to the center of the matrix; for instance, the first and last regions are homologous. (C) Similarity of functional brain networks across subjects (bar chart with means and associated standard deviations), for the average subject (diamonds), and when adding homotopic connections (circles) (left). Network maps for the average subject and empirical FC, as well as for FC simulated using either the SAR model with original SC or the SARh.
(EPS)

Figure S4 **Effect of time on performance.** Predictive power of computational models as a function of the time-window length for each subject (graphs) and model (color).
(EPS)

Figure S5 **Exploration of the parameter space for the Fitzhugh-Nagumo model.** (Left) Phase diagrams (i.e., *x*-*y* plane) for an uncoupled model (*k* = 0) over various parameter values of *α* and *β*. The model operate mostly in an oscillatory regime for the range of parameter values investigated. (Right) Predictive power as a function of *α* and *β*. The black dot represents the parameter set used in our simulations, while the black square corresponds to the values from [28]. The values used in our simulations gave rise to higher predictive power than the parameters values from [28]. In any case, for the range of parameters considered, the predictive power always remained lower than that obtained with a SAR model.
(EPS)

Figure S6 **Effect of velocity on predictive power.** Predictive power as a function of the coupling strength and velocity values in generative models. Black dots represent values used for subsequent simulations. These simulations show that the predictive power is little influenced by velocity. In any case, for the range of parameters considered, the predictive power also always remained lower than that obtained with a SAR model.
(EPS)

Figure S7 **Effect of the hemodynamic model.** Predictive power for all connections and when restricted to intra/interhemispheric, direct/indirect connections. For each type of connections and each model, we represented the predictive power for the average subject computed using the BOLD signal (diamonds, solid line) or using the neuronal activity (circles, dashed line). Of note, the prediction differs slightly from that of the Figure 1 due to the stochastic component of most models at each run.
(EPS)

Text S1 **Data and preprocessing.**
(PDF)

Text S2 **Computational models.**
(PDF)
