# Supplementary material for: Relating Structure and Function in the Human Brain: Relative Contributions of Anatomy, Stationary Dynamics, and Non-stationarities
Source: PLoS Comput Biol. 2014 Mar 20;10(3):e1003530. doi: 10.1371/journal.pcbi.1003530 (PMC3961181; doi:10.1371/journal.pcbi.1003530)
Supplement: Text S1 — Data and preprocessing. (PDF) [file pcbi.1003530.s008.pdf]

S1 Data and preprocessing

Resting-state fMRI series were recorded using a single-shot, gradient-recalled echo-planar imaging sequence (repetition time TR: 3290 ms; echo time TE: 31 ms; $1.5 \times 1.5 \times 2.5$ mm^3 voxels; 46 contiguous slices). Two hundreds rs-fMRI volumes were acquired, for a total duration of 11 min. The subjects were instructed to remain eyes closed and to reduce any mental effort. DWI data were recorded using a single-shot, echo planar imaging sequence (TR: 13 s; TE: 121 ms; 2 mm^3 isotropic voxels; 68 contiguous slices). Fifty encoding directions with $b = 1000$ s/mm^2 and a non-weighted image were acquired for each subject. A three dimensional, T_1-weighted, magnetization prepared rapid gradient-echo volume was also acquired during the same scanning session (TR: 2.3 ms; TE: 2.98 ms; 1.1 mm^3 isotropic voxels).

Rs-fMRI data were preprocessed using the SPM5 software (`http://www.fil.ion.ucl.ac.uk/spm/software/spm5/`). For each subject, the first 4 volumes were discarded to allow for T_1 equilibration, and the remaining 196 volumes were corrected for slice-timing and head motion. Excessive motion (greater than 3 mm or 3 degrees) was not present in any of the subjects' scans. The resulting data were then spatially smoothed using an isotropic 6 mm full-width-at-half-maximum Gaussian kernel. DWI images were corrected for eddy-current distortions using FSL, release 4.1 (`http://www.fmrib.ox.ac.uk/fsl/`) [1]. Spatial normalization between rs-fMRI and DWI data, on the one hand, and the anatomical volume, on the other hand, were computed for each subject using FSL and linear transformations (combination of 3 translations, 3 rotations and 1 scale factor). For visualization purposes, nonlinear spatial normalization to the standard space of the Montreal Neurological Institute (MNI) was also computed from the T_1-weighted anatomical volume of each subject.

Regions of interest

The T_1-weighted anatomical volume of each subject was parcellated using the Freesurfer software (`http://surfer.nmr.mgh.harvard.edu/`) [2] and the procedure described in [3]. The procedure divided the brain into cortical and sub-cortical gray and white matter. A labeled cortical surface from an average template brain was projected onto the individual cortical surfaces. For each subject, this step provided a partition of the brain cortical surface and sub-cortical volume into 74 and 6 regions, respectively, for each hemisphere. Finally, these regions were projected on the rs-fMRI and DWI subjects' spaces using the linear transformations previously calculated.

Anatomical wiring connections

To quantify structural connectivity, a probabilistic white matter fiber tracking method [4] implemented in FSL was used to track all possible connections between all pairs of regions. For every voxel of the white matter we initiated 500 fiber samples. Starting points and initial fiber orientations were randomly selected. Fibers were grown in the two opposite directions; propagation step was set to 0.5 mm and maximal fiber curvature to 80° (no anisotropy constraint). Fiber tracking was stopped when a sample reached the cortical surface or a sub-cortical volume. An index of structural connectivity between two regions was then defined as the proportion of fiber samples connecting these two regions per unit surface. This index was further divided by the average fiber length to reduce bias towards longer fibers. This structural connectivity index allowed to build a 160-by-160 individual structural connectivity matrix $\mathbf{D}$ for each subject, D_{rs} being the structural connectivity index from region r to region s, with no self-connections ($D_{rr} = 0$). $\mathbf{D}$ was then thresholded at 0.001; supra-threshold values were kept as such. Similarly, we built a 160-by-160 matrix $\mathbf{L}$ of between-region fiber lengths for each subject, used to estimate conduction delays in certain computational models.

BOLD signal

The time series of all voxels within a given region were spatially averaged to form the representative signal of that region. To remove spurious sources of variance, linear and quadratic drifts, motion parameters, averaged ventricular, white matter and global brain signals were regressed out. The global signal was defined as the spatial average of all time series within the brain (where fMRI data was available). Ventricular and white matter signals were defined as the spatial averages of the time courses corresponding to voxels belonging to the regions that had the highest probability in the probabilistic maps of ventricles and white matter, respectively. Finally, time series were low-pass filtered (< 0.1 Hz) [5, 6].

References

[1] S M Smith, M Jenkinson, M W Woolrich, C F Beckmann, T E J Behrens, H Johansen-Berg, P R Bannister, M De Luca, I Drobnjak, D E Flitney, R K Niazy, J Saunders, J Vickers, Y Zhang, N De Stefano, J M Brady, and P M Matthews. Advances in functional and structural MR image analysis and implementation as FSL. *Neuroimage*, 23:208–19, 2004.

[2] B Fischl, A Van der Kouwe, C Destrieux, E Halgren, F Segonne, D H Salat, E Busa, L J Seidman, J Goldstein, D Kennedy, V Caviness, N Makris, B Rosen, and A M Dale. Automatically parcellating the human cerebral cortex. *Cerebral Cortex*, 14:11–22, 2004.

[3] P Hagmann, L Cammoun, X Gigandet, R Meuli, C Honey, V Wedeen, and O Sporns. Mapping the structural core of human cerebral cortex. *PLoS Biology*, 6:e159, 2008.

[4] T E J Behrens, H Johansen-Berg, S Jbabdi, M F S Rushworth, and M W Woolrich. Probabilistic diffusion tractography with multiple fibre orientations: What can we gain? *Neuroimage*, 34:144–55, 2007.

[5] M Fox, D Zhang, A Snyder, and M Raichle. The global signal and observed anticorrelated resting state brain networks. *Journal of Neurophysiology*, 101:3270–83, 2009.

[6] K Van Dijk, T Hedden, A Venkataraman, K Evans, S Lazar, and R Buckner. Intrinsic functional connectivity as a tool for human connectomics: Theory, properties, and optimization. *Journal of Neurophysiology*, 103:297–321, 2010.
