# Supplementary material for: Relating Structure and Function in the Human Brain: Relative Contributions of Anatomy, Stationary Dynamics, and Non-stationarities
Source: PLoS Comput Biol. 2014 Mar 20;10(3):e1003530. doi: 10.1371/journal.pcbi.1003530 (PMC3961181; doi:10.1371/journal.pcbi.1003530)
Supplement: Text S2 — Computational models. (PDF) [file pcbi.1003530.s009.pdf]

S2 Computational models

The **SAR model** expresses the BOLD signal $\mathbf{y} = (y_i)$ as a linear combination of the fluctuations within other regions [1, 2]

$$y_i = k \sum_{j \neq i} D_{ij} y_j + \sigma \nu_i. \quad (1)$$

k is a parameter of spatial autoregression, σ is the noise level, and the ν_i's stand for uncorrelated realizations of Gaussian noise with zero mean and unit variance. $\mathbf{y}$ is further assumed to be multivariate normal with zero mean and covariance matrix that can be calculated from Eq. (1) as

$$\sigma^2 (\mathbf{I} - k\mathbf{D})^{-1} (\mathbf{I} - k\mathbf{D})^{-\mathrm{t}}, \quad (2)$$

where $\mathbf{I}$ stands for the identity matrix and "$-\mathrm{t}$" is the inverse of the regular matrix transposition. In the simulations, σ was set to one.

The **Wilson-Cowan model** explores large ensembles of excitatory (E) and inhibitory (I) neurons using a mean-field approach [3, 4]. The dynamics is governed by the following equations:

$$\begin{aligned} \tau_E \frac{\partial E_i(t)}{\partial t} &= -E_i(t) + \phi \left[I_b + k \sum_j D_{ij} E_i(t - \tau_{ij}) - I_i(t) \right] + \sigma \nu_i \\ \tau_I \frac{\partial I_i(t)}{\partial t} &= -I_i(t) + \phi \left[\omega_I E_i(t) \right] + \sigma \nu_i, \end{aligned}$$

with $D_{ii} = \omega_+/k$, and where τ_E and τ_I correspond to the time constant (or scale) of the excitatory and inhibitory population, respectively. ω_I is the action level of the excitatory population on the inhibitory population, ω_+ the self-retroaction of excitatory population. I_b is a diffuse spontaneous background input. τ_{ij} is the propagation delay between regions i and j, based on the average fiber tract length between regions scaled by axonal velocity, v, i.e., $\tau_{ij} = L_{ij}/v$. ν_i is a random fluctuating input accounting for sources of biophysical variability and was defined as in the SAR model. The transfer function ϕ accounts for the saturation of firing rates in neuronal populations and is modeled by a sigmoid: $\phi(x) = c[1 - e^{-a(x-b)}]^{-1}$. Parameters were set to: $\tau_I = \tau_E = 20$ ms; $\omega_I = 0.5$; $\omega_+ = 0.5$; $v = 10$ m/s; $I_b = 0$; $\sigma = 0.25$; $a = 5$; $b = 0$; and $c = 2$.

The **rate model** is a simplification of the Wilson-Cowan system [5], where inhibitory populations and saturation function ϕ are removed

$$\tau \frac{\partial u_i(t)}{\partial t} = -u_i(t) + k \sum_{j \neq i} D_{ij} u_i(t - \tau_{ij}) + \sigma \nu_i.$$

Here we have $\tau = 20$ ms; $v = 10$ m/s; and $\sigma = 0.25$.

The **Kuramoto model** is composed of a set of coupled oscillators

$$\frac{\partial \phi_i(t)}{\partial t} = 2\pi f_i + k \sum_{j \neq i} D_{ij} \sin\left[\phi_i(t) - \phi_j(t - \tau_{ij})\right] + \sigma \nu_i,$$

where θ_i and f_i stand for the phase and intrinsic frequency of region i. We set $f_i = 60$ Hz; $v = 10$ m/s; and $\sigma = 1.25$ rd.

The **Fitzhugh-Nagumo model** is composed of two nested variables [6]

$$\begin{aligned} \tau_x \frac{\partial x_i(t)}{\partial t} &= g[x_i(t), y_i(t)] + k \sum_{j \neq i} D_{ij} x_j(t - \tau_{ij}) + \sigma \nu_i \\ \tau_y \frac{\partial y_i(t)}{\partial t} &= h[x_i(t), y_i(t)] + \sigma \nu_i, \end{aligned}$$

where

$$\begin{aligned} g[x_i(t), y_i(t)] &= \gamma x_i(t) - \frac{x_i^3(t)}{3} - y_i(t) \\ h[x_i(t), y_i(t)] &= -\beta y_i(t) + x_i(t) + \alpha, \end{aligned}$$

$\tau_x = 20$ ms; $\tau_y = 100$ ms; $v = 10$ m/s; $\sigma = 0.25$; $\alpha = 0.8$; $\beta = 0.6$; and $\gamma = 1$.

The **neural-mass model** is a nonlinear biophysical model of neuronal dynamics relying on the Hodgkin-Huxley model [7].

The main dynamical variables are the mean membrane potential of excitatory and inhibitory populations (V and Z, respectively), which are governed by the conductance of sodium, potassium and calcium ions, and the passive conductance of leaky ions, g_{ion}. The total current flow across pyramidal cell membranes is given by:

$$\begin{aligned}
\frac{\partial V_i(t)}{\partial t} &= -m_{\text{Ca}}\left[g_{\text{Ca}} + r_{\text{NMDA}} a_{ee} k \sum_j D_{ij} Q_{V_j}\right](V_i(t) - V_{\text{Ca}}) \\
&\quad - \left[g_{\text{Na}} m_{\text{Na}} + a_{ee} k \sum_j D_{ij} Q_{V_j}\right](V_i(t) - V_{\text{Na}}) \\
&\quad - g_{\text{K}} W (V_i(t) - V_{\text{K}}) - g_{\text{L}}(V_i(t) - V_{\text{L}}) \\
&\quad + a_{ie} Z Q_{Z_i} + a_{ne} I_\delta \\
\frac{\partial Z_i(t)}{\partial t} &= b\left[a_{ii} V_i Q_{V_i} + a_{ni} I_\delta\right],
\end{aligned}$$

with $D_{ii} = (1-k)/k$, and where m_{ion} and V_{ion} are the fractions of open ion channels and the Nernst potential for that ion species, respectively. For large ion channels population, the fraction of open ion channels is given by the sigmoid-shaped neural activation function,

$$m_{\text{ion}} = \frac{1}{2}\left[1 + \tanh\left(\frac{V - T_{\text{ion}}}{\delta_{\text{ion}}}\right)\right],$$

except for the potassium channels that decay exponentially,

$$\frac{\partial W}{\partial t} = \frac{\phi(m_{\text{K}} - W)}{\tau}.$$

Q_V and Q_Z represent the average firing-rates of excitatory and inhibitory neurons,

$$Q_X = \frac{Q_{X_{\max}}}{2}\left[1 + \tanh\left(\frac{X - X_T}{\delta_X}\right)\right].$$

I_δ corresponds to nonspecific subcortical excitation. a_{xy} scales the x-to-y synaptic strength and r_{NMDA} corresponds to the number of NMDA receptors. Parameters are set to values taken from [8].

The **spiking neurons model** models each region as a biophysically realistic attractor consisting of mutually interconnected populations of excitatory pyramidal neurons and inhibitory neurons [9]. This type of attractor network of spiking neurons is a dynamical system with an intrinsic tendency to settle in stationary states, also called attractors, typically characterized by a stable pattern of firing activity. Small perturbations may induce transitions between different stable attractors. Mean-field approximation yields a set of nonlinear equations of average firing rates of each population. For full details on the model definition and parameters, see [10].

Optimization of coupling parameter

All models had as inputs a normalized form of the structural connectivity matrix as well as the value for a parameter that represented the coupling strength between regions. To limit the influence of these inputs, we performed an optimization step prior to data simulation. For each model, we generated data with different matrix normalization strategies and values for the coupling parameter and kept the configuration that maximized predictive power. For normalization, we considered 2 approaches: spectral normalization and row normalization [11]. Spectral normalization consists of dividing the matrix by its spectral radius, i.e. the largest absolute value of its eigenvalues. Row normalization imposes that the matrix rows sum to 1 [2]. For the coupling parameter, known bounds were used to constrain the optimization when available (SAR, rate).

Numerical details of simulations

All simulations were performed in Matlab (The MathWorks Inc., Natick, MA), except for the spiking neurons model which is implemented in language C. The SAR model gives a closed form for the covariance matrix [see Eq. (2)] that we used to directly compute the FC predicted by this model. Dynamical models were simulated at a sampling frequency of 10 kHz. Simulations of the rate, Wilson-Cowan, Kuramoto, and Fitzhugh-Nagumo models relied on the Euler integration scheme, while Matlab ordinary differential equations solver was used for the neural-mass model. The resulting data were then

downsampled to a time resolution of 1 ms. The data corresponding to the first 20 s were discarded from the analysis to avoid transient dynamics, resulting in 8 min of simulated brain activity. Simulated rs-fMRI BOLD signal, sampled at 2 Hz, was obtained from neuronal activity by means of the Balloon-Windkessel hemodynamic model [12]. We computed three runs with random initial conditions and averaged the corresponding FCs to improve stability.

References

[1] P Whittle. On stationary processes in the plane. *Biometrika*, 41:434–49, 1954.

[2] G Tononi, O Sporns, and GM Edelman. A measure for brain complexity: relating functional segregation and integration in the nervous system. *Proceedings of the National Academy of Sciences of the United States of America*, 91:5033–7, 1994.

[3] H R Wilson and J D Cowan. Excitatory and inhibitory interactions in localised populations of model neurons. *Biophysical Journal*, 12, 1972.

[4] G Deco, V Jirsa, A R McIntosh, O Sporns, and R Kotter. Key role of coupling, delay, and noise in resting brain fluctuations. *Proceedings of the National Academy of Sciences of the United States of America*, 106:10302–7, 2009.

[5] R Galán. On how network architecture determines the dominant patterns of spontaneous neural activity. *PLoS ONE*, 3:e2148, 2008.

[6] A Ghosh, Y Rho, A R McIntosh, R Kotter, and V Jirsa. Noise during rest enables the exploration of the brain's dynamic repertoire. *PLoS Computational Biology*, 4:e1000196, 2008.

[7] M Breakspear, J R Terry, and K J Friston. Modulation of excitatory synaptic coupling facilitates synchronization and complex dynamics in a biophysical model of neuronal dynamics. *Network: Computation in Neural Systems*, 14:703–32, 2003.

[8] C J Honey, O Sporns, L Cammoun, X Gigandet, J-P Thiran, R Meuli, and P Hagmann. Predicting human resting-state functional connectivity from structural connectivity. *Proceedings of the National Academy of Sciences of the United States of America*, 106:2035–40, 2009.

[9] Gustavo Deco and Viktor K Jirsa. Ongoing cortical activity at rest: Criticality, multistability, and ghost attractors. *The Journal of Neuroscience*, 32:3366–75, 2012.

[10] Gustavo Deco, Viktor Jirsa, and Anthony McIntosh. Emerging concepts for the dynamical organization of resting-state activity in the brain. *Nature Reviews Neuroscience*, 12:43–56, 2011.

[11] L Barnett, C L Buckley, and S Bullock. Neural complexity and structural connectivity. *Physical Review E*, 79:051914, 2009.

[12] K J Friston. Dynamic causal modelling. *Neuroimage*, 19:1273–302, 2003.
